# Retraction Note: Taurine plays a beneficial role against cadmium-induced oxidative renal dysfunction

**DOI:** 10.1007/s00726-024-03390-w

**Published:** 2024-03-20

**Authors:** Prasenjit Manna, Mahua Sinha, Parames C. Sil

**Affiliations:** Department of Chemistry, Bose Institute, 93/1, Acharya Prafulla Chandra Road, Kolkata, 700009 West Bengal India

**Retraction Note: Amino Acids (2009) 36:417–428** 10.1007/s00726-008-0094-x

The Editor-in-Chief has retracted this article. Concerns have been raised regarding the data presented in Fig. 4. Specifically:

- Figure 4b appears to show repetitive features;
- The data in Fig. 4 were later reused in the authors' other publications (Roy et al. 2009; Manna and Sil 2012; Ghosh et al. 2010) to represent different treatment groups.

The authors have stated that the images were reused in the other articles by mistake, and are correctly included in this article. However, they have been unable to explain the repetitive features in Fig. 4b. The Editor-in-Chief therefore no longer has confidence in the presented data.

Prasenjit Manna and Parames C. Sil agree to this retraction. The Publisher has not been able to obtain a current email address for Mahua Sinha.
